# Correlation between triglyceride glucose index and coronary plaque: An observational study

**DOI:** 10.1097/MD.0000000000039576

**Published:** 2024-09-13

**Authors:** Haiyan Jia, Weifeng Zhang, Shengqi Jia, Jun Zhang, Zesheng Xu, Zhanwen Xu, Yaqin Li

**Affiliations:** a Department of Cardiology, Cangzhou Central Hospital, Tianjin Medical University, Tianjin, China; b Department of Cardiology, Affiliated Hospital of Hebei University, Baoding, China; c Department of Cardiology, Second Hospital of Hebei Medical University.

**Keywords:** triglyceride glucose index, type 2 diabetes mellitus, vulnerable plaque

## Abstract

The association between the triglyceride-glucose (Tyg) index and coronary plaque in patients with coronary heart disease remains unclear. This study aimed to investigate the relationship between Tyg index and coronary plaque under different levels of blood glucose metabolism. This retrospective study included patients with coronary artery disease who underwent coronary angiography and OCT between January 1, 2023 and January 1, 2024, and ultimately collected 232 coronary plaques. All patients were grouped according to the median Tyg index (T1 group 7.71 ≤ TyG index ≤ 9.13; T2 group 9.14 ≤ TyG index ≤ 10.99). The thickness of plaque fiber cap was measured under OCT, and the plaques were divided into vulnerable plaque and non-vulnerable plaque. The status of glucose metabolism is divided into non-diabetic and diabetic. Baseline data analysis showed that there were significant differences in clinical and biological characteristics between the T1 and T2 groups (*P* < .05). Logistic regression analysis showed that T2 group was significantly associated with vulnerable plaques compared with T1 group (odds ratio [OR]: 2.638; 95% confidence interval [CI] 1.548–4.494; *P* < .001). The OR of Tyg index was 2.175 (95% CI 1.409–3.357; *P* < .001). Receiver operating characteristic showed that the area under ROC curve (AUC) was 0.727 (95% CI 0.663–0.792; *P* < .001), the best cutoff value was 9.23, the sensitivity was 60%, and the specificity was 81%. In diabetic patients, there was a statistically significant correlation between Tyg index and coronary vulnerable plaque (OR: 3.273; 95% CI 1.240–8.636, *P* < .05). Triglyceride glucose index is a good predictor of coronary vulnerable plaque.

## 1. Introduction

Coronary heart disease (CHD) is a chronic cardiovascular disease caused by coronary arteriosclerosis. Due to aging, urbanization, obesity and unhealthy lifestyle, the incidence and mortality of coronary heart disease are on the rise.^[1,2]^ Type 2 diabetes is recognized as a common complication that affects the progression of coronary heart disease, and there is growing evidence that glycemic management can have a significant impact on cardiovascular outcomes.^[3]^ The risk of adverse cardiovascular events was significantly increased in CHD patients with diabetes.^[4,5]^ Coronary angiography can accurately assess the degree of coronary stenosis for diagnosing coronary heart disease. OCT can correctly identify coronary vulnerable plaques. However, many patients miss the tests due to high expense and invasiveness. The triglyceride glucose (Tyg) index is a combination of fasting blood glucose (FPG) and triglyceride (TG) calculations and is considered to be a representative biomarker of insulin resistance (IR).^[6]^ Many studies have shown that Tyg is closely related to atherosclerosis.^[7–11]^ In addition, an elevated Tyg index has potential value in the early identification of individuals at high risk for cardiovascular disease.^[12]^ However, the association between the triglyceride-glucose (Tyg) index and coronary plaque in patients with coronary heart disease remains unclear. The influence of glucose metabolism status also needs to be further studied.

The purpose of this study was to investigate the relationship between Tyg index and coronary vulnerable plaque in patients with coronary heart disease, as well as the influence of different glucose metabolism status on the relationship between Tyg index and coronary vulnerable plaque. In addition, this study determined the accuracy of Tyg index in detecting coronary plaque instability.

## 2. Methods

### 2.1. Study population

This retrospective study included patients with coronary heart disease who underwent coronary angiography and OCT between January 1, 2023 and January 1, 2024, and ultimately collected 232 coronary plaques. The following patients were excluded: younger than 35 years or older than 75 years; suffering from tumor, infectious disease, autoimmune disease; Incomplete clinical data, poor OCT image quality cannot analyze plaque characteristics. The study has been approved by the Ethics Committee of Cangzhou Central Hospital (2023-331-01 [Y]) and the Ethics Committee of the Affiliated Hospital of Hebei University (HDFYLL-KY-2023-080), and registered in the Chinese Clinical Trials Registry (ChiCRT2300072737). Independent clinical researchers observed the trial and accumulated the data.

### 2.2. Research method

Anthropometric data such as weight, height and body mass index were recorded, as well as personal information such as age, gender, smoking history and alcohol consumption history. The blood samples tested were fasting venous blood collected from all participants by medical professionals in the early morning. The levels of FPG, HbA1c, TG, total cholesterol (TC), high density lipoprotein cholesterol (HDL-C) and low density lipoprotein cholesterol (LDL-C) were measured by automatic hematology analyzer.

### 2.3. Definition

Tyg index calculation formula was defined as: Ln[TG(mg/dL) × FPG (mg/dL)/2].^[13]^ Body mass index (BMI) is calculated by dividing your weight by the square of your height and is expressed in kg/m^2^. Coronary heart disease is defined as lumen stenosis of ≥ 50% in at least one major coronary artery (left anterior descending branch, left circumflexal branch, and right coronary artery). The degree of coronary stenosis under OCT is described as follows: mild stenosis < 50%, moderate stenosis ≥ 50–80%, and severe stenosis ≥ 80%. The plaque features of patients were examined by OCT, and the plaques with fiber cap < 65 µm and lipid arc ≥ 2 quadrants were defined as vulnerable plaques.^[14,15]^ According to the American Diabetes Association (ADA) 2016 guidelines,^[16]^ diabetes is defined as FPG ≥ 7.0 mmol/L or HbA1c ≥ 6.5%. Normal blood glucose regulation was defined as FPG < 6 mmol/L or HbA1c < 6%.

### 2.4. Statistical analysis

Continuous data are expressed as mean ± standard deviation or median and interquartile intervals. Categorical variables are expressed as percentages. The differences between groups were calculated using the χ^2^ test and *t* test. Mann–Whitney test or Kruskal–Wallis test for continuous variables. Logistic regression analysis was used to calculate odds ratio (OR) and 95% confidence interval (CI) to examine the correlation between Tyg index and coronary vulnerable plaque in patients with coronary heart disease. The area under the curve (AUC) and 95% CI of the receiver Operating characteristic (ROC) curve were calculated to determine the accuracy of the Tyg index in detecting coronary vulnerable plaques. A *P* value < .05 was considered statistically significant. All statistical analyses were performed using SPSS 24.0.

## 3. Result

### 3.1. Baseline information

There were 232 participants in this study, and the mean age was 58 ± 10 years, and 79.3% were male. All patients were grouped according to the median Tyg index (T1 group 7.71 ≤ TyG index ≤ 9.13; Group T2 9.14 ≤ TyG index ≤ 10.99). Compared with T1 group, BMI, white blood cell count, lymphocyte count, TG, AIP, creatinine, FPG and C-reactive protein (CRP) were higher in T2 group, but HDL-C was lower (*P* < .05). There were significant differences in the history of alcohol consumption, hypertension, diabetes and renal insufficiency between T1 and T2 groups. There were significant differences in the degree of coronary artery stenosis, the proportion of vulnerable plaque and the proportion of macrophages between T1 and T2 groups (*P* < .05; Table 1).

**Table 1:** Clinical and biological characteristics of the T1 and T2 group.*

Items	T1 (<9.13)	T2 (≥9.13)	F/Z/ χ^2^	*P* †
Age (yr)	59.3 ± 10.9	58.4 ± 9.2	1.293	.539
BMI	24.9 ± 2.7	25.9 ± 3.3	3.860	.016
WBC (×10^9^)	6.7 ± 2.3	7.3 ± 1.7	5.958	**.031**
HGB (g/L)	140.9 ± 13.3	140.3 ± 12.9	1.485	.701
PLT (×10^9^/L)	226.7 ± 50.0	225.9 ± 78.0	13.090	.929
Neutrophil count (×10^9^/L)	5.0 ± 1.1	4.7 ± 1.4	4.117	.620
Lymphocyte count (×10^9^/L)	1.7 ± 0.5	1.9 ± 0.7	16.904	**.009**
TC (mmol/L)	4.4 ± 1.2	4.4 ± 1.1	0.610	.868
TG (mmol/L)	1.3 ± 0.4	2.4 ± 0.8	49.581	**0**
LDL-C (mmol/L)	2.8 ± 0.8	2.8 ± 0.9	3.453	.855
HDL-C (mmol/L)	1.2 ± 0.4	1.0 ± 0.2	6.279	**0**
AIP	0.20 (0.01, 0.26)	0.31 (0.15, 0.49)	−5.967	**0**
cTnI (ng/mL)	0.01 (0.01, 0.36)	0.01 (0.01, 0.06)	−0.773	.440
Cr (µmol/L)	74.0 (62.5, 83.0)	67.0 (59.0, 81.0)	−1.919	**.042**
UA (µmol/L)	337.1 ± 82.1	323.0 ± 98.6	2.800	.240
BNP (pg/mL)	63.0 (22.5, 115.5)	50.0 (22.9, 118)	−0.370	.788
EF (%)	61.4 ± 9.0	61.3 ± 5.2	8.921	.907
FBG	5.2 (5.0, 7.0)	8.0 (7.0, 12.0)	−0.858	**0**
Tyg index	8.6 ± 0.3	9.7 ± 0.5	3.348	**0**
SAA	9.0 ± 3.3	9.3 ± 3.5	0.002	.459
CRP	4.8 (4.1, 5.7)	5.2 (5.0, 7.0)	−2.394	**.017**
HCY	12.1 (10.0, 15.4)	11.4 (8.6, 16.2)	−0.725	.469
Sex (Man)	92 (84.4)	92 (74.8)	3.250	.071
Smoking history	83 (76.1)	81 (65.9)	2.955	.086
Drinking history	40 (36.7)	19 (15.4)	13.761	**0**
Hypertension	50 (45.9)	89 (72.4)	16.990	**0**
Diabetes	20 (18.3)	48 (39.0)	11.923	**0**
Renal dysfunction	5 (4.6)	17 (13.8)	5.741	**.017**
Personal history of cerebral infarction	10 (9.2)	13 (10.6)	0.126	.723
Degree of coronary stenosis			6.196	.045
Mild coronary stenosis	12 (75)	4 (25)		
Moderate coronary stenosis	58 (47.5)	64 (52.5)		
Severe coronary stenosis	39 (41.5)	55 (58.5)		
Vulnerable plaque	38 (34.9)	72 (58.5)	12.990	**0**
Calcified plaque	60 (73.4)	100 (81.3)	2.077	.150
Ruptured plaque	19 (17.4)	16 (13.0)	0.883	.348
Macrophage	58 (53.2)	93 (75.6)	12.759	**0**
Red blood clots	11 (10.1)	9 (7.3)	0.565	.452
White blood clots	14 (12.8)	18 (14.6)	0.156	.693
Microchannel	46 (42.2)	58 (47.2)	0.573	.449
Cholesterol crystal	60 (55.0)	70 (56.9)	0.082	.775

AIP = atherogenic index of plasma, BMI = body mass index, BNP = brain natriuretic peptide, CI = confidence interval, CRP = C-reactive protein, cTnI = cardiac troponin I, EF = ejection fraction, FPG = fasting blood glucose, HCY = homocysteine, HDL-C = high density lipoprotein cholesterol, HGB = hemoglobin, LDL-C = low density lipoprotein cholesterol, PLT = platelet, OR = odds ratio, SAA = serum amyloid A, TC = total cholesterol, TG = triglyceride, Tyg = triglyceride-glucose, UA = uric acid, WBC = white blood cells.

* Plus–minus values are means ± SD.

† *P* values were calculated with the use of Fisher’s exact test for categorical variables, the Wilcoxon rank-sum test for AIP/cTnI/Cr/ BNP/ FBG/CRP/HCY, and Student *t* test for the remaining continuous variables.

Bold denotes statistical significant values having *P* < .05.

### 3.2. Comparsions between the vulnerable plaque and non-vulnerable plaque group

Compared with non-vulnerable plaque group, FPG, triglyceride glucose index and CRP in vulnerable plaque group were higher (*P* < .05). There were significant differences in gender, alcohol consumption history, hypertension history and renal insufficiency history between the non-vulnerable plaque group and the vulnerable plaque group. The proportion of macrophages, red thrombi and red thrombi in vulnerable plaque was higher than that in non-vulnerable plaque (*P* < .05; Table 2).

**Table 2:** Clinical and biological characteristics of vulnerable plaque and non-vulnerable plaque.*

Items	Non-vulnerable plaque	Vulnerable plaque	F/Z/ χ^2^	*P* †
Age (yr)	57.9 ± 10.3	59.9 ± 0.9	0.044	.135
BMI	25.4 ± 3.1	25.5 ± 3.0	0.019	.773
WBC (×10^9^)	7.1 ± 2.1	6.9 ± 1.9	0.353	.562
HGB (g/L)	140.7 ± 11.8	140.5 ± 14.4	0.274	.869
PLT (×10^9^/L)	234.9 ± 59.0	216.6 ± 69.6	1.212	**.031**
Neutrophil count (×10^9^/L)	4.6 ± 1.9	5.1 ± 1.7	1.121	.481
Lymphocyte count (×10^9^/L)	1.8 ± 0.5	1.9 ± 0.7	7.391	.381
TC (mmol/L)	4.4 ± 1.1	4.4 ± 1.1	1.087	.700
TG (mmol/L)	2.0 ± 0.7	2.1 ± 0.8	0.627	.690
LDL-C (mmol/L)	2.8 ± 0.9	2.7 ± 0.9	0.724	.628
HDL-C (mmol/L)	1.1 ± 0.4	1.0 ± 0.3	2.652	.242
AIP	0.22 (0.04, 0.33)	0.21 (0.11, 0.32)	−0.180	.857
cTnI (ng/mL)	0.01 (0.01, 0.16)	0.01 (0.01, 0.09)	−0.824	.410
Cr (µmol/L)	72.0 (61.0, 83.0)	69.0 (59.0, 81.0)	−1.311	.190
UA (µmol/L)	336.7 ± 87.0	321.9 ± 94.7	1.396	.218
BNP (pg/mL)	42.7 (22.4, 119.1)	63.6 (32.8, 115.3)	−0.951	.341
EF (%)	61.4 ± 8.0	61.3 ± 6.1	1.908	.999
FBG	5.1 (5.0, 8.0)	8.0 (6.0, 12.0)	−5.377	**0**
Tyg index	9.0 ± 0.6	9.3 ± 0.6	0.310	**0**
SAA	8.4 ± 2.4	10.0 ± 3.3	0.057	.811
CRP	4.8 ± 1.1	6.0 ± 2.1	22.861	**0**
HCY	12.0 (9.9, 14.8)	11.8 (9.6, 15.0)	−0.053	.958
Sex (Man)	89 (73.0)	95 (86.4)	6.342	**.012**
Smoking history	78 (63.9)	86 (78.2)	5.667	**.017**
Drinking history	34 (27.9)	25 (22.7)	0.806	.369
Hypertension	62 (50.8)	77 (70.0)	9.860	**.030**
Diabetes	30 (24.6)	38 (34.5%)	2.767	.096
Renal dysfunction	3 (2.5)	19 (17.3)	14.789	**0**
Personal history of cerebral infarction	9 (7.4)	14 (12.7)	1.854	.173
Degree of coronary stenosis			6.196	.045
Mild coronary stenosis	9 (56.3)	7 (43.8)	2.116	.347
Moderate coronary stenosis	69 (56.6)	53 (43.4)		
Severe coronary stenosis	44 (46.8)	50 (53.2)		
Vulnerable plaque	72 (59.0)	79 (71.8)	4.172	**.041**
Calcified plaque	3 (2.5)	17 (15.5)	12.401	**0**
Ruptured plaque	7 (5.7)	25 (22.7)	14.042	**0**
Macrophage	54 (44.3)	50 (45.5)	0.033	.855
Red blood clots	68 (55.7)	62 (56.4)	0.009	.924

AIP = atherogenic index of plasma, BMI = body mass index, BNP = brain natriuretic peptide, CI = confidence interval, CRP = C-reactive protein, cTnI = cardiac troponin I, EF = ejection fraction, FPG = fasting blood glucose, HCY = homocysteine, HDL-C = high density lipoprotein cholesterol, HGB = hemoglobin, LDL-C = low density lipoprotein cholesterol, PLT = platelet, OR = odds ratio, SAA = serum amyloid A, TC = total cholesterol, TG = triglyceride, Tyg = triglyceride-glucose, UA = uric acid, WBC = white blood cells.

* Plus–minus values are means ± SD.

† *P* values were calculated with the use of Fisher’s exact test for categorical variables, the Wilcoxon rank-sum test for AIP/cTnI/Cr/BNP/FBG/ HCY, and Student *t* test for the remaining continuous variables.

Bold denotes statistical significant values having *P* < .05.

### 3.3. Relationship between Tyg index and vulnerable plaques

**Figure 1.**
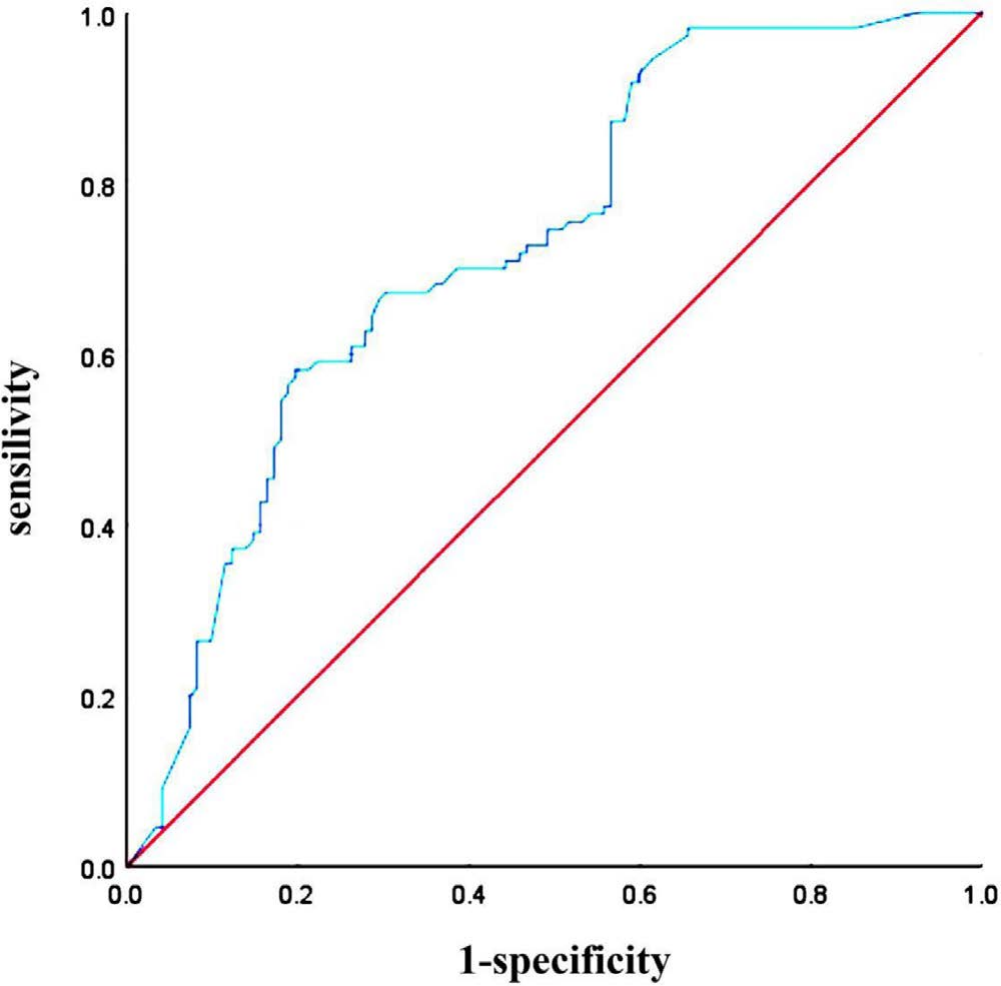
Receiver operating characteristic (ROC) curves analysis of Tyg index for coronary vulnerable plaques. ROC = receiver operating characteristic.

The relationship between vulnerable plaques and various risk factors was evaluated by binary logistic regression. Compared with non-vulnerable plaques, smoking history, history of hypertension, history of renal insufficiency, HCY, CRP, SSA, fasting blood glucose, Tyg index were significantly correlated with vulnerable coronary plaques (*P* < .05). When Tyg index was used as categoric variable, Patients in T2 had a 2.638 times greater risk of developing vulnerable plaques than those in T1 (95% CI 1.548–5.797, *P* < .001; Table 3). Tyg index was significantly positively correlated with coronary vulnerable plaques (OR: 2.175, 95% CI 1.409–3.357; *P* < .001). As shown in Table 4, after the logistic regression model was constructed, Tyg index was significantly correlated with coronary vulnerable plaques when analyzed as a continuous variable before and after multiple adjustments (*P* < .05). Receiver operating characteristic (ROC) curves analysis of Tyg index for coronary vulnerable plaques showed that the AUC of Tyg index was 0.727 (95% CI 0.663–0.792; *P* < .001), the best cutoff value was 9.23, the sensitivity was 60%, and the specificity was 81% (Table 5, Fig. 1).

**Table 3:** Relationship between coronary vulnerable plaques and risk factors.

Variable	Vulnerable plaques			
	OR (95% CI)		β	*P*
Sex				
Women	Reference			
Man	1.824 (0.944, 3.522)		0.611	.074
Age	1.020 (0.994, 1.047)		0.02	.134
BMI	1.012 (0.931, 1.101)		0.012	.771
Smoking				
No	Reference			
Yes	2.021 (1.127, 3.623)		0.704	.018
Drinking				
No	Reference			
Yes	0.761 (0.419, 1.382)	−0.273		.37
Hypertension				
No	Reference			
Yes	2.258 (1.315, 3.877)		0.815	.003
Diabetes				
No	Reference			
Yes	1.619 (0.916, 2.860)		0.482	.097
Personal history of cerebral infarction				
No	Reference			
Yes	1.813 (0.759, 4.416)		0.605	.178
Renal dysfunction				
No	Reference			
Yes	8.282 (2.378, 18.845)		2.114	.001
TC	0.957 (0.765, 1.196)		−0.044	.699
TG	0.959 (0.782, 1.176)		−0.042	.689
HCY	1.174 (1.101, 1.251)		0.16	<.001
CRP	1.389 (1.161, 1.663)		0.329	<.001
SAA	1.154 (1.065, 1.249)		0.143	<.001
FPG	1.190 (1.100, 1.288)		0.174	<.001
Tyg index	2.175 (1.409, 3.357)		0.777	<.001
T1	Reference			
T2	2.638 (1.548, 5.797)		1.183	<.001

CI = confidence interval, BMI = body mass index, CRP = C-reactive protein, FPG = fasting blood glucose, HCY = homocysteine, OR = odds ratio, SSA, TC = total cholesterol, TG = triglyceride, Tyg = triglyceride-glucose.

**Table 4:** Correlation analysis of triglyceride glucose index and vulnerable plaque.

Variable		Vulnerable plaques				
	OR (95% CI)*	*P*	OR (95% CI)†	*P*	OR (95% CI)‡	*P*
Tyg index	2.175 (1.409, 3.357)	0	3.264 (1.838, 5.797)	0	3.273 (1.240, 8.636)	.017

CI = confidence interval, BMI = body mass index, CRP = C-reactive protein, HCY = homocysteine, OR = odds ratio, SSA, TC = total cholesterol, TG = triglyceride, Tyg = triglyceride-glucose.

* Model a: Uncorrected.

† Model b: Corrected for age, sex, and BMI.

‡ Model c: Corrects gender, age, BMI, smoking history, drinking history, hypertension history, diabetes history, cerebral infarction history, renal insufficiency history, TC, TG, HCY, CRP, SSA, fasting blood glucose.

**Table 5:** ROC curve result of Tyg index for coronary vulnerable plaques.

Standard error*	Sig†	95% CI	
		Lower limits	Upper limit
0.033	<0.001	0.663	0.792

ROC = receiver operating characteristic. Tyg = triglyceride-glucose.

* Under the nonparametric assumption.

† Null hypothesis: true area = 0.5.

### 3.4. Relationship between Tyg index and coronary vulnerable plaque in non-diabetic and diabetic

After multivariate adjustment, no statistical difference was found between Tyg index and coronary vulnerable plaque in non-diabetic patients (*P* > .05). However, in diabetic patients, Tyg index was significantly correlated with coronary vulnerable plaques, with statistical difference (OR: 3.273; 95% CI 1.240–8.636, *P* < .05; Table 6).

**Table 6:** The relationship between Tyg index and vulnerable plaque in different glucose metabolic states.

Glucose metabolic status	Variable		Vulnerable plaques				
		OR (95% CI)*	*P*	OR (95% CI)†	*P*	OR (95% CI)‡	*P*
Diabetes mellitus	Tyg	1.701 (0.465, 6.223)	.422	2.057 (0.509, 8.313)	.312	3.273 (1.240, 8.636)	.017
Nondiabetic	Tyg	1.477 (0.651, 3.354)	.351	1.851 (0.786, 4.362)	.159	1.417 (0.961, 9.318)	.054

CI = confidence interval, BMI = body mass index, CRP = C-reactive protein, HCY = homocysteine, OR = odds ratio, SSA, TC = total cholesterol, TG = triglyceride, Tyg = triglyceride-glucose.

* Model a: Uncorrected.

† Model b: Corrected for age, sex, and BMI.

‡ Model c: Corrects gender, age, BMI, smoking history, drinking history, hypertension history, diabetes history, cerebral infarction history, renal insufficiency history, TC, TG, HCY, CRP, SSA, fasting blood glucose.

## 4. Discussion

With the popularization of knowledge about coronary heart disease, although the identified cardiovascular risk factors (dyslipidemia, hypertension, and pro-coagulant state) have been effectively treated, there are still a large number of unexplained CV risks, which may be related to metabolic syndrome such as obesity caused by changes in residents’ lifestyle and improvement in diet structure. Insulin resistance is the main core of metabolic inflammation and the central link in the pathogenesis of many metabolic diseases.^[17,18]^ Studies have found that vascular diseases caused by insulin resistance have begun to appear in early life, and children and adolescents with insulin resistance are prone to complicated vascular damage.^[18]^ Chen et al^[19]^ used Mendelian randomization analysis to find a causal relationship between insulin resistance and the risk of coronary artery disease, myocardial infarction and ischemic stroke, providing genetic evidence to support that insulin resistance may lead to an increased risk of cardiovascular disease. In recent years, the triglyceride-glucose index (Tyg), calculated by Ln [fasting triglyceride (mg/dL) × fasting glucose (mg/dL)/2], has been considered as a novel alternative to insulin resistance.^[20]^ Current research in China, South Korea, Spain and other countries found Tyg index was significantly associated with the development of type 2 diabetes.^[21–23]^ Studies have found that insulin resistance often appears before the traditional risk factors for atherosclerosis such as diabetes, dyslipidemia, and hypertension. Before the clinical onset of type 2 diabetes, asymptomatic patients experience a period of time characterized by insulin resistance, and latent vascular dysfunction begins to develop at this stage. Therefore, patients with T2D are at increased cardiovascular risk long before the disease is diagnosed. After adjusting for traditional cardiovascular risk factors, the degree of atherosclerosis in subjects with insulin resistance was higher than that in subjects with normal glucose tolerance.^[24]^ Sanchez-Ingol^[25]^ study included 5014 patients and found that cardiovascular disease risk increased with increased Tyg index, independent of other known cardiovascular risk factors (such as age, sex, body mass index, smoking, hypertension, T2DM, etc). A study in Tianjin, China, showed that Tyg index was significantly associated with the risk of multi-vessel coronary heart disease. After adjusting for confounding factors, the risk of multiple-vessel coronary artery disease increased 2.28 times in the high TyG group.^[26]^ A large number of basic studies have proved that the abnormal conduction of insulin signals in the state of insulin resistance leads to endothelial dysfunction, which leads to vasodilation collapse and cell inflammation, and promotes the deposition of large amounts of low-density lipoprotein cholesterol in the blood vessel wall. Through the scavenger receptor on its surface, circulating monocytes phagocytose oxidized low-density lipoprotein cholesterol with high affinity, differentiate into arterial macrophages, and then become foam cells, forming atherosclerotic lipid streaks. Insulin resistance can promote plaque formation.^[27]^ Insulin resistance further induces oxidative stress, which leads to fiber cap thinning and plaque necrosis, and the cellular and non-cellular components of blood circulation directly contact the highly thrombogenic lipid core components, forming a cascade reaction to form thrombosis, resulting in stenosis or even occlusion of the infarct-related coronary arteries.^[28,29]^ Diabetic patients had higher levels of fat-rich plaques and higher rates of macrophage accumulation. In non-criminal plaques, the diabetic group had a larger maximum lipid arc and a thinner fiber cap thickness, suggesting that the plaques on both criminal and non-criminal lesions in diabetic patients had more vulnerable characteristics than in non-diabetic patients, indicating that diabetic patients had higher panvascular plaque instability.^[30]^ Studies have analyzed combined biomarkers of inflammation, lipids, and clotting, looking for biomarker patterns associated with thin-cap atherosclerotic plaques and “high-risk plaques.” Based on the findings of high sensitivity C-reactive protein, plasminogen activator inhibitor-1, fibrinogen, IL-6, homocysteine and amyloid A levels increased, and high density lipoprotein cholesterol (HDL) and cholic acid levels decreased, 2 different TCFA complex models were established. Both composite models showed high detection accuracy in TCFA patients (area under the curve [AUC] of model a: 0.883, and area under the curve [AUC] of model b: 0.875, *P* < .001). In addition, creatinine, highly sensitive C-reactive protein, fibrinogen, tumor necrosis factor-α, IL-6, homocysteine, amyloid A, HDL, prothrombin, and bile acids can be used to detect “high-risk plaque” patients. The detection accuracy of the 2 composite models was higher in patients with “high-risk plaques” (AUC of model a was 0.925, AUC of model b was 0.947, *P* < .001)).^[31]^ The calculation method of Tyg index is simple, the cost is low, and most medical units can carry out it, which is very suitable for clinical application in primary hospitals. At present, the correlation between Tyg index and coronary vulnerable plaque in patients with coronary heart disease needs to be further studied, and the influence of different glucose metabolism status on the correlation also needs to be further studied. This study showed that compared with T1 group (7.71 ≤ TyG index ≤ 9.13 group), TG, creatinine, FPG and CRP in T2 group (9.14 ≤ TyG index ≤ 10.99) were higher, but HDL-C was lower (*P* < .05). There were significant differences in the history of alcohol consumption, hypertension, diabetes and renal insufficiency between T1 and T2 groups. There were significant differences in the degree of coronary artery stenosis, the proportion of vulnerable plaque and the proportion of macrophages between T1 and T2 groups (*P* < .05). Compared with non-vulnerable plaque group, FPG, triglyceride glucose index and CRP in vulnerable plaque group were higher (*P* < .05). Studies have shown that compared with non-chronic kidney disease patients, patients with chronic kidney disease have a higher lipid index of coronary plaque, and a higher incidence of plaque rupture and calcified plaque. Multiple linear regression models showed that lower glomerular filtration rate was an independent risk factor for higher plaque lipid index.^[32]^ It was consistent with the results of this study that the vulnerable plaque group had higher prevalence of hypertension and renal insufficiency. The proportion of macrophages, red thrombi and red thrombi in vulnerable plaque was higher than that in non-vulnerable plaque (*P* < .05). Meanwhile, this study showed that compared with T1 group (7.71 ≤ TyG index ≤ 9.13), T2 group (9.14 ≤ TyG index ≤ 10.99) was significantly correlated with vulnerable plaques (odds ratio [OR]: 2.638; 95% confidence interval [CI] 1.548–4.494; *P* < .001). The OR of Tyg index was 2.175 (95% CI 1.409–3.357; *P* < .001). The area under ROC curve (AUC) of Tyg index for coronary vulnerable plaques was 0.727 (95% CI 0.663–0.792; *P* < .001), the best truncation value is 9.23. In diabetic patients, there was a statistically significant correlation between Tyg index and coronary vulnerable plaque (OR: 3.273; 95% CI 1.240–8.636, *P* < .05). Therefore, Tyg index can help us identify high-risk groups of CHD early, and assist doctors in risk stratification, diagnosis and treatment of patients. Tyg index has the advantages of low cost, simple operation and high application value in clinical and epidemiological research. Further study of the relationship between Tyg index and coronary heart disease in the future can provide more economical and feasible methods for primary doctors to prevent and treat coronary heart disease and other chronic diseases.

## Author contributions

**Investigation:** Shengqi Jia.

**Visualization:** Shengqi Jia.

**Writing – original draft:** Haiyan Jia, Weifeng Zhang, Jun Zhang, Zesheng Xu, Zhanwen Xu, Yaqin Li.

**Writing – review & editing:** Haiyan Jia, Weifeng Zhang, Jun Zhang, Zesheng Xu.
